# *Cannabis* Use Is Associated With Increased Levels of Soluble gp130 in Schizophrenia but Not in Bipolar Disorder

**DOI:** 10.3389/fpsyt.2020.00642

**Published:** 2020-07-02

**Authors:** Attila Szabo, Ibrahim A. Akkouh, Thor Ueland, Trine Vik Lagerberg, Ingrid Dieset, Thomas Bjella, Pål Aukrust, Stephanie Le Hellard, Anne-Kristin Stavrum, Ingrid Melle, Ole A. Andreassen, Srdjan Djurovic

**Affiliations:** ^1^ NORMENT, Institute of Clinical Medicine, University of Oslo, and Division of Mental Health and Addiction, Oslo University Hospital, Oslo, Norway; ^2^ Department of Medical Genetics, Oslo University Hospital, Oslo, Norway; ^3^ Research Institute of Internal Medicine, Oslo University Hospital Rikshospitalet, Oslo, Norway; ^4^ Section of Clinical Immunology and Infectious Diseases, Oslo University Hospital, Rikshospitalet, Oslo, Norway; ^5^ K.G. Jebsen Inflammatory Research Center, University of Oslo, Oslo, Norway; ^6^ K.G. Jebsen Thrombosis Research and Expertise Center, University of Tromsø, Tromsø, Norway; ^7^ NORMENT, Department of Clinical Science, University of Bergen, Bergen, Norway; ^8^ Center for Medical Genetics and Molecular Medicine, Haukeland University Hospital, Bergen, Norway

**Keywords:** *Cannabis*, immune modulation, sgp130, IL-6 trans-signaling, schizophrenia, bipolar disorder

## Abstract

The complex effects of plant cannabinoids on human physiology is not yet fully understood, but include a wide spectrum of effects on immune modulation. The immune system and its inflammatory effector pathways are recently emerging as possible causative factors in psychotic disorders. The present study aimed to investigate whether self-administered *Cannabis* use was associated with changes in circulating immune and neuroendocrine markers in schizophrenia (SCZ) and bipolar disorder (BD) patients. A screening of 13 plasma markers reflecting different inflammatory pathways was performed in SCZ (n = 401) and BD patients (n = 242) after subdividing each group into *Cannabis* user and non-user subgroups. We found that i) soluble gp130 (sgp130) concentrations were significantly elevated among *Cannabis* users in the SCZ group (p = 0.002) after multiple testing correction, but not in BD. ii) Nominally significant differences were observed in the levels of IL-1RA (p = 0.0059), YKL40 (p = 0.0069), CatS (p = 0.013), sTNFR1 (p = 0.031), and BDNF (p = 0.020), where these factors exhibited higher plasma levels in *Cannabis* user SCZ patients than in non-users. iii) These differences in systemic levels were not reflected by altered mRNA expression of genes encoding sgp130, IL-1RA, YKL40, CatS, sTNFR1, and BDNF in whole blood. Our results show that *Cannabis* self-administration is associated with markedly higher sgp130 levels in SCZ, but not in BD, and that this phenomenon is independent of the modulation of peripheral immune cells. These findings warrant further investigation into the potential IL-6 trans-signaling modulatory, anti-inflammatory, neuroimmune, and biobehavioral-cognitive effects of *Cannabis* use in SCZ.

## Introduction

The psychoactive and medicinal properties of the *Cannabis indica* plant, a species of the genus *Cannabis* of Cannabaceae family, have been recognized for millennia for its therapeutic value in inflammation, pain, and rheumatic diseases (1). The biologically active cannabinoids can be classified into two major groups based on their psychotropic effect. The psychoactive delta-9 tetrahydrocannabinol (Δ9-THC or THC) and the non-psychoactive cannabidiol (CBD) are the two most widely studied cannabinoids with potential therapeutic value. However, beyond their effects on immunity and inflammation, cannabinoids have also been shown to modulate neuronal development, as well as to influence the general cytoarchitecture of the brain by regulating axon and dendrite growth, synaptic dynamics, and pruning (2). This latter phenomenon has recently been linked to their capacity to increase the levels of neurotrophins in the mammalian CNS, such as brain-derived neurotrophic factor (BDNF) (3, 4). It has also been associated with potential adverse effects with regard to cognition, behavior, respiratory, and cardiovascular disorders (5, 6).

Schizophrenia (SCZ) and bipolar disorder (BD) are severe mental illnesses which pose a substantial burden on the global community by greatly affecting the mortality, life quality, and costs of patient care (7). Although both have a strong genetic component, details of their pathophysiology and etiology is not yet known and the understanding of the underlying disease mechanisms will expectedly be crucial for developing new and effective therapies (8). The immune system and its inflammatory effector pathways and elements are recently emerging as possible contributing factors in psychotic disorders (9). Inflammatory cytokines may alter brain functions in multiple ways, and can pass the blood-brain-barrier through leaky regions or *via* passive and active transport mechanisms. Abnormal immune activation and dysregulated inflammatory responses have been suggested to be involved in the pathophysiology of psychosis, and represent potential underlying mechanisms in both SCZ and BD (10–12). Plant cannabinoids have been shown to exert a wide spectrum of effects on human immunity ranging from anti-inflammation to neuroimmune modulation (13). THC and CBD are known to have disparate influences on SCZ and BD symptoms, and probably on their pathophysiology as well.

Despite the intensive research efforts in the past decades, the complex effects of plant cannabinoids on human physiology, including effects on the immune system, are not yet fully understood, partly due to law regulations of plant-based exocannabinoids in many countries. Since medical *Cannabis* has already entered mainstream medicine, it is necessary for the medical community to make efforts assessing the possible risks and benefits of plant cannabinoids in order to provide reliable and competent clinical care. In this study, we aimed to investigate the effects of *Cannabis* consumption (users versus nonusers) on circulating inflammatory, immune, and neuroendocrine markers in SCZ and BD patients. The following biomarkers were selected as they reflect different (sub)parts of the inflammatory network, also because they are stable factors with relatively long biological half-life that provide reliable information with regard to long-term changes in immune system responses: interleukin 1 receptor antagonist (IL-1RA), soluble glycoprotein 130 (sgp130, the natural inhibitor of soluble interleukin-6 receptor trans-signaling responses), and soluble tumor necrosis factor receptor 1 (sTNFR1), which are general markers of inflammation; the neutrophil markers myeloperoxidase (MPO) and cathepsin S (CatS); YKL40 (also known as Chitinase-3-like protein 1 or CHI3L1), which is a marker of monocyte/macrophage activation; chemokine C-X-C motif ligand 16 (CXCL16), pentraxin 3 (PTX3), osteoprotegerin (OPG), von Willebrand factor (vWF), and activated cell adhesion molecule (ALCAM), which are markers of vascular inflammation; Parkinson disease protein 7 (PARK7) and BDNF, which are markers of neuroendocrine modulation in various neuropsychiatric disorders. We and others have previously shown that some of these markers are dysregulated in psychosis (e.g., IL-1Ra, OPG, sTNFR1, vWf, and sgp130), while some are related to neuroinflammation (e.g., CXCL16 and ALCAM) and can be modulated by cannabinoids (4–6, 14–16). The aim of this study is to investigate if self-administered *Cannabis* use is associated with alterations in immune and neuroendocrine plasma markers in SCZ and BD. Here, we report the modulatory potential of *Cannabis* on circulating immune and neuroendocrine markers in SCZ in a disease-specific manner.

## Materials and Methods

### Setting and Participants

The current study is part of the ongoing Thematically Organized Psychosis (TOP) study, which includes participants from hospitals in the Oslo region, Trondheim, and Southeast regional hospitals in Norway. Information about recruitment procedures, inclusion and exclusion criteria, and clinical assessments for the TOP study as a whole have been described in detail in previous reports (16, 17). All participants have given written consent and the study was approved by the Norwegian Scientific Ethical Committees and the Norwegian Data Protection Agency. The authors assert that all procedures contributing to this work comply with the ethical standards of relevant guidelines and regulations.

### Sample Characteristics

The primary study sample of the present work consisted of 242 bipolar disorder patients (BD; 164 type I, 66 type II, 12 not otherwise specified) and 401 schizophrenia spectrum patients (SCZ; 296 schizophrenia, 77 schizoaffective, 28 schizophreniform). All participants underwent a clinical examination that included diagnostic interviews based on Structured Clinical Interview in DSM-IV axis I Disorders (SCID-1) and structured assessments of clinical symptoms, BMI, use of psychotropic medication, smoking habits, alcohol consumption, and illicit substance use. Diagnostic evaluation was performed by trained psychologists and psychiatrists, all of whom participated regularly in diagnostic meetings supervised by professors in psychiatry. The main inclusion criteria were confirmed diagnosis of either SCZ spectrum disorder or BD according to the Diagnostic and Statistical manual of Mental Disorders (DSM)-IV, age between 18 and 65, and ability to give informed written consent. The main exclusion criteria were clinically significant brain injury, neurological disorder, and any substance use other than *Cannabis* within the last 6 months prior to blood sampling. Patients with ongoing infection, autoimmune disorders or cancer were also excluded. *Cannabis* was self-administered in the user cohorts and “*Cannabis* use” was defined as any use in the last 6 months prior to blood sampling. Samples were subjected to high sensitivity plasma EIA and Illumina microarray analyses. Substance use was documented through interviews, urine samples, the Clinical Drug Use Scale (DUS) and the Clinical Alcohol Use Scale (AUS) (18, 19). Substance use disorders were diagnosed using the SCID-E module. All accessible information in each case was examined to avoid false positive and false negative substance users. The study sample also included 613 healthy controls (284 females, 329 males; mean age 33.4). Since sufficient data on illegal drug use was not available for the controls, they were not included in the statistical analyses, but they were used to determine the average plasma concentrations in healthy subjects for the examined biomarkers.

### Measurement of Inflammatory and Neuroendocrine Markers in Plasma

Blood samples from both disease cohorts were obtained at Oslo University Hospital (OUH) following the same standardized procedures in accordance with the EUSTAR guidelines on biobanking (20). Peripheral blood samples were collected in EDTA tubes and were centrifuged at >2,100 g at room temperature within 30 min. Plasma aliquots were then stored at −80°C until assayed. Circulating YKL40, CatS, sTNFR1, BDNF, sgp130, IL-1RA, Alcam, MPO, CXCL16, Park7, vWF, OPG, and PTX3 levels were analyzed in duplicate using commercially available antibodies (all from R&D Systems, Minneapolis, MN, USA; except IL-1Ra, Peprotech, RockyHill, NJ, USA) in a 384 format using a combination of a SELMA (Jena, Germany) pipetting robot and a BioTek (Winooski, VT, USA) dispenser/washer. Absorption was read at 450 nm with wavelength correction set to 540 nm using an ELISA plate reader (Bio-Rad, Hercules, CA, USA). Intra- and inter-assay coefficients of variation were <10% for all EIAs.

### RNA Expression Analysis

Blood samples for mRNA expression measurements were collected in Tempus Blood RNA Tubes (Life Technologies Corporation). Total RNA was extracted with the TEMPUS 12-Port RNA Isolation Kit (Applied Biosystems) and ABI PRISM 6100 Nucleic Acid PrepStation (Applied Biosystems) according to the manufacturer’s protocol. Gene expression analysis of immune-related genes was performed with Illumina HumanHT-12 v4 Expression BeadChip (Illumina, Inc.). Multidimensional scaling and hierarchical clustering were used for regular quality control, including sample quality measurements and removal of outliers, as well as removal of multiple batch effects. This was followed by log2-transformation. If more than one probe for a single gene were available, only the constitutive probe targeting all gene transcripts was used. In total, six mRNA probes targeting six *Cannabis*-associated immune marker genes were examined. More details on microarray pre-processing and quality control have previously been reported (21).

### Statistical Analyses

Within each diagnostic category (BD and SCZ), patients were divided into two groups based on whether they had used any *Cannabis* within the last 6 months prior to blood sampling (users) or not (non-users). To identify significant differences between users and non-users in clinical metrics of interest, Student’s two-sided t-test was used for numerical variables (the Wilcoxon rank sum test was used for non-parametric variables) and Pearson’s Chi-squared test was used for categorical variables. To investigate whether plasma concentrations of inflammatory and neuroendocrine biomarkers were related to *Cannabis* use within each diagnostic group, separate multiple linear regression analyses were performed for BD and SCZ. The predictor variable was *Cannabis* status (non-user vs. user) and the response variable was circulating immune marker concentrations. All analyses were adjusted for sex, age, BMI, smoking status (yes/no), alcohol consumption, and the use of four different types of medication (antipsychotics, anticonvulsants, antidepressants, and lithium). Medication doses were standardized prior to analysis by converting them to Defined Daily Dosages (DDD) in accordance with the guidelines from the World Health Organization Collaborating Center for Drug Statistics Methodology (https://www.whocc.no/). The Bonferroni procedure was used to adjust the p-values for the number of statistical tests performed. Because of the unbalanced design of our datasets, we log2-transformed any immunological marker with severely non-normal residuals or unequal variances between groups. To examine whether *Cannabis* status was associated with gene expression levels of the significant inflammatory markers, a separate but partially overlapping sample of 249 BD patients (180 type I, 54 type II, 15 not otherwise specified) and 383 SCZ patients (270 schizophrenia, 78 schizoaffective, 35 schizophreniform) was used (**Supplementary Figure S2**, **Supplementary Table S1**). The same statistical procedures as described above were followed. All analyses were performed in R 3.4.1.

## Results

Demographic and clinical variables are summarized in **Table 1**. In both diagnostic groups, age was strongly associated with *Cannabis* use, and *Cannabis* users were on average 6.1 years younger than non-users. Among SCZ patients, associations were also found between *Cannabis* use and BMI (p = 6.54e-5), tobacco smoking (p = 3.93e-9), alcohol consumption (p = 6.91e-7), and age of onset of psychotic symptoms (1.34e-4). Interestingly, BD patients who smoked *Cannabis* experienced less negative symptoms than patients without recent *Cannabis* use (p = 0.009). In the SCZ group, *Cannabis* use was associated with an increased risk of psychotic symptoms in total (p = 0.039), which is in agreement with the recent literature (5, 15). There were no significant differences in the use of antipsychotics, antiepileptics, or lithium in any of the disease groups, but SCZ patients who smoked *Cannabis* used less antidepressants than non-smokers (p = 0.027).

**Table 1:** Demographic and clinical characteristics of schizophrenia (SCZ) and bipolar disorder (BD) patients in the immune marker sample grouped according to recent *Cannabis* use (User) or no recent *Cannabis* use (Non-user).

	SCZ patients						BD patients					
	Non-user		User				Non-user		User			
	n = 341		n = 60				n = 205		n = 37			
	Mean	SD	Mean	SD	Test statistic	p-value	Mean	SD	Mean	SD	Test statistic	p-value
Age	31.9	10.5	26.0	7.7	t = −5.18	1.15e-6**	35.5	12.6	29.2	9.3	t = −3.63	5.67e-4**
Sex, female (n, %)	161	47.2	16	26.7	χ^2^ = 7.42	0.006*	125	61.0	18	48.6	χ^2^ = 1.49	0.222
Education (years)	12.9	2.9	12.0	2.1	t = −2.75	0.007*	14.5	3.0	13.7	3.0	t = −1.41	0.165
BMI	27.0	5.6	24.5	3.7	t = −4.17	6.54e-5**	25.6	4.5	25.4	4.7	t = −0–31	0.756
Tobacco use, yes (n, %)	161	47.2	52	86.7	χ^2^ = 34.66	3.93e-9**	100	48.8	26	70.3	χ^2^ = 4.97	0.026*
Alcohol units 6 months (MD, range)	10	0–1456	65	0–1098	W = 5769.5	6.91e-7**	52	0–1820	117	2–2184	W = 2404.5	0.001*
Age of onset (years)	24.5	8.6	20.8	6.1	t = −3.96	1.35e-4**	22.8	9.9	21.4	7.7	t = −1.01	0.318
PANSS positive	15.2	5.4	17.1	5.3	t = 2.60	0.011*	10.2	3.9	11.7	4.1	t = 2.10	0.041*
PANSS negative	15.8	6.3	16.7	6.8	t = 0.90	0.372	10.4	3.8	9.0	2.7	t = −2.68	0.009*
PANS general	31.9	9.0	34.4	8.3	t = 2.04	0.044*	26.1	6.8	25.2	6.2	t = −0.72	0.477
PANSS total	63.0	17.2	68.1	17.5	t = 2.10	0.039*	46.6	11.9	45.4	10.6	t = −0.60	0.548
IDS	18.8	12.9	14.5	11.7	t = −1.88	0.068	16.1	11.5	16.0	12.0	t = −0.07	0.946
YMRS	5.1	5.0	6.3	5.6	t = 1.33	0.189	3.7	5.1	4.5	5.1	t = 0.87	0.387
GAF Symptoms	42.7	11.3	40.1	12.1	t = −1.54	0.127	56.6	12.5	56.2	10.1	t = −0.19	0.846
GAF Function	43.7	11.0	40.2	9.8	t = −2.48	0.015*	53.2	12.7	53.0	11.8	t = −0.09	0.925
Antipsychotics (DDD)	1.23	1.22	1.44	0.99	t = 1.47	0.146	0.53	0.76	0.34	0.50	t = −1.90	0.061
Anticonvulsants (DDD)	0.084	0.26	0.057	0.24	t = −0.79	0.432	0.32	0.52	0.24	0.41	t = −1.09	0.282
Antidepressants (DDD)	0.47	0.85	0.28	0.55	t = −2.24	0.027*	0.51	0.89	0.42	0.78	t = −0.66	0.509
Lithium (DDD)	0.026	0.18	0.017	0.13	t = −0.48	0.630	0.24	0.50	0.20	0.46	t = −0.52	0.604

**p < 0.001, *p < 0.05. BMI, Body Mass Index; PANSS, Positive and Negative Syndrome Scale; IDS, Inventory of Depression Scale; YMRS, Young Mania Rating Scale; GAF, Global Assessment of Functioning; MD, Median; DDD, Defined Daily Dose.

### Significant Association Between *Cannabis* Use and sgp130 Levels

**Figure 1:**
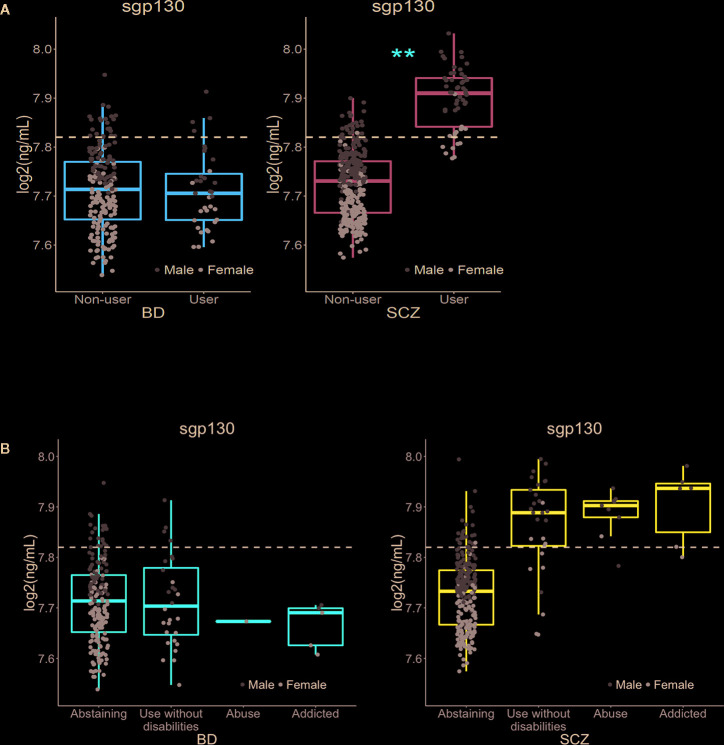
Effect of *Cannabis* use on soluble gp130 levels in bipolar disorder and schizophrenia patients, and associations between frequency of *Cannabis* use and sgp130 levels in BD and SCZ. **(A)** *Cannabis* use was significantly associated with increased levels of soluble gp130 in schizophrenia (SCZ) but not in bipolar disorder (BD) patients. The dotted lines represent the mean plasma level of gp130 in healthy controls (n = 613). The average gp130 level in BD patients, in both *Cannabis* users and non-users, and in non-user SCZ patients was below the average level found in healthy controls. However, SCZ patients who have used *Cannabis* had an elevated gp130 level above the level for healthy controls. As the dot plots indicate, *Cannabis* use had a comparable up-regulating effect in both males and females. **Bonferroni-adjusted p-value < 0.01. **(B)** Patterns of *Cannabis* use during the last 6 months before blood sampling were assessed with the Clinician Drug Use Scale (CDUS). No significant difference in sgp130 concentration was found between the different subgroups of *Cannabis* users in the SCZ group (F(2,42) = 0.44, p = 0.65). The lack of a dose-response relationship between *Cannabis* exposure and sgp130 concentration may be due to the lack of detailed temporal resolution in the *Cannabis* data. Since sgp130 levels are expected to depend on the time interval that has passed since the last exposure to *Cannabis*, the inability to account for temporal differences may mask the dose-dependent effect of *Cannabis* on sgp130 concentrations.

We first compared the levels of 13 stable plasma biomarkers involved in immune and neuroendocrine regulation (YKL40, CatS, sTNFR1, BDNF, sgp130, IL-1RA, Alcam, MPO, CXCL16, Park7, vWF, OPG, and PTX3) in the *Cannabis* user versus non-user groups among BD and SCZ patients. The circulating levels of sgp130 were significantly higher among *Cannabis* users in the SCZ group after adjustment for the number of tests performed (p = 0.002; **Figure 1A**, **Table 2**). The log2 mean difference was 0.175, which translates to an increase of 27.3 ng/ml (12.9%) in absolute concentration. Non-user SCZ patients had a mean sgp130 level below the mean concentration found in healthy controls, but this deficiency was completely reversed in the *Cannabis* group (**Figure 1A**). Females had on average lower sgp130 levels than males, and this difference was generally not affected by *Cannabis* use, indicating that *Cannabis* had similar effects on sgp130 levels in both genders. To further explore the nature of the sgp130 association in SCZ, patients were grouped according to the severity of *Cannabis* use. However, no dose-dependent relationship was found between *Cannabis* use and sgp130 (F(2, 42) = 0.44, p = 0.65), which could be explained by the lack of detailed temporal resolution in the *Cannabis* data (**Figure 1B**). Although tobacco smoking was strongly correlated with *Cannabis* use, the duration and frequency of tobacco smoking did not have a significant effect on sgp130 concentration (p = 0.75; **Supplementary Figure S1**). No significant difference in sgp130 levels between users and non-users was observed in the BD group (p = 0.87; **Figure 1**, **Table 2**). All BD patients, independent of *Cannabis* status, had suboptimal sgp130 levels comparable to the levels found in non-user SCZ patients (**Figure 1**).

**Table 2:** Associations between inflammatory and neuroendocrine markers in plasma and Cannabis use in schizophrenia and bipolar disorder patients.

Immune marker	SCZ patients						BD patients					
			Non-user		User				Non-user		User	
	p-value Nominal	p-value Bonferroni	Mean	SD	Mean	SD	p-value Nominal	p-value Bonferroni	Mean	SD	Mean	SD
sgp130	1.62e-4**	2.11e-3	7.724	0.067	7.899	0.063	0.866	1.000	7.712	0.081	7.712	0.079
IL-1RA	5.91e-3*	0.077	7.783	0.424	8.241	0.304	0.255	1.000	7.711	0.500	7.285	0.572
YKL40	6.93e-3*	0.090	5.231	0.236	5.480	0.178	0.279	1.000	5.395	0.404	5.341	0.324
CatS	0.013*	0.165	2.346	0.216	2.496	0.195	0.392	1.000	2.276	0.157	2.307	0.149
BDNF	0.020*	0.255	2.230	0.174	2.534	0.142	0.259	1.000	2.254	0.181	2.072	0.197
sTNFR1	0.031*	0.400	0.839	0.240	0.984	0.127	0.145	1.000	0.831	0.176	0.882	0.197
Park7	0.182	1.000	2.314	0.196	2.201	0.154	0.947	1.000	2.131	0.000	2.111	0.208
MPO	0.247	1.000	8.020	0.190	8.280	0.190	0.418	1.000	8.201	0.382	7.827	0.407
Alcam	0.327	1.000	38.619	2.694	38.205	2.104	0.756	1.000	38.880	2.224	37.160	2.454
vWF	0.379	1.000	6.335	0.226	6.214	0.167	0.352	1.000	6.126	0.308	6.356	0.348
OPG	0.400	1.000	0.420	0.112	0.418	0.085	0.082	1.000	0.485	0.148	0.305	0.156
PTX	0.500	1.000	1.576	0.250	1.833	0.205	0.600	1.000	1.530	0.238	1.476	0.222
CXCL16	0.701	1.000	4.060	0.162	4.099	0.150	0.059	0.769	3.972	0.156	4.156	0.121

**Significantly associated after Bonferroni correction at ⍺ = 0.05. *Nominally associated at p < 0.05. The means and standard deviations (SD) shown are for the fitted values from the full regression model.

### Nominally Significant *Cannabis* Associations

**Figure 2:**
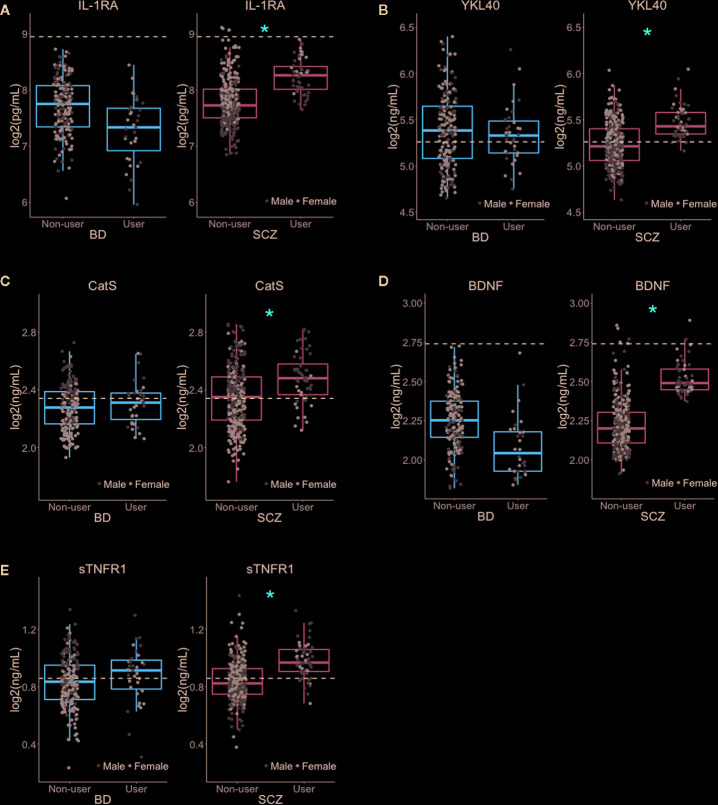
Nominally significant associations between *Cannabis* use and inflammatory and neuroendocrine markers **(A–E)**. *Cannabis* use had a nominally significant association (at p < 0.05) with five immune and neuroendocrine markers in SCZ but not in BD patients. All markers had higher plasma levels in *Cannabis* users than in non-users. The dotted lines represent the average concentration in healthy controls (n = 613). *Nominal p-value < 0.05.

In the SCZ group, nominally significant associations (i.e., associations that did not survive multiple testing correction) were found between *Cannabis* exposure and five inflammatory markers: IL-1RA (p = 0.0059), YKL40 (p = 0.0069), CatS (p = 0.013), sTNFR1 (p = 0.031), and the neurotrophin BDNF (p = 0.020; **Figure 2**, **Table 2**). Although *Cannabis* users had increased levels of all five markers compared to non-users, IL-1RA and BDNF levels were still below the average concentration found in healthy controls. Females had generally higher levels of IL-1RA and lower levels of CatS than males, while no gender differences were seen for YKL40, BDNF, and sTNFR1 (**Figure 2**).

### Associations Between *Cannabis* Use and mRNA Expression

To examine if the observed changes in systemic markers could be attributed to the modulation of circulating immune cells by *Cannabis* exposure, we tested whether *Cannabis* status was also associated with the mRNA expression levels of IL6ST, IL1RN, CHI3LI, CTSS, BDNF, and TNFRSF1A genes encoding sgp130, IL-1RA, YKL40, CatS, BDNF, and sTNFR1, respectively. No significant associations were found for any of the genes in BD or SCZ patients (**Supplementary Figure S3**; **Supplementary Table S2**). Neither did we find any significant associations for mRNA expression of the rest of the genes of our screening platform (MPO, YKL40, CXCL16, PTX3, OPG, vWF, ALCAM, and PARK7) in our patient cohorts (data not shown).

## Discussion

The aim of the present study was to investigate whether self-administered *Cannabis* use was associated with changes in circulating immune and neuroendocrine markers in SCZ and BD. We performed a neuroimmune screening of 13 plasma markers in SCZ and BD patients, subdividing each group into *Cannabis* user and non-user subgroups. We found that sgp130 concentrations were markedly elevated among *Cannabis* users in the SCZ but not in the BD group. We also found indications of increased levels of IL-1RA, YKL40, CatS, sTNFR1, and BDNF among *Cannabis* using SCZ patients, but these differences were not significant after Bonferroni correction. These results may therefore reflect individual differences in *Cannabis*-related inflammatory functions (IL-1RA, YKL40, CatS, and sTNFR1) within and between the sample groups rather than being SCZ or BD-specific biological features. Interestingly, the two major cannabinoids THC and CBD have recently been shown to increase the levels of neurotrophins in the mammalian CNS including BDNF, and thus can contribute to increased neuroplasticity and improved cognitive performance (3, 4). Our results corroborate these findings and suggest a potential neurotrophin modulating effect of *Cannabis* in SCZ.

Changes in the plasma concentrations of sgp130, IL-1RA, YKL40, CatS, sTNFR1, and BDNF were not accompanied by corresponding changes at the gene expression level in whole blood samples indicating limited contribution from peripheral immune cell modulation, but could potentially reflect the involvement of other tissues, such as the liver, vascular endothelium, or glial cells.

**Figure 3:**
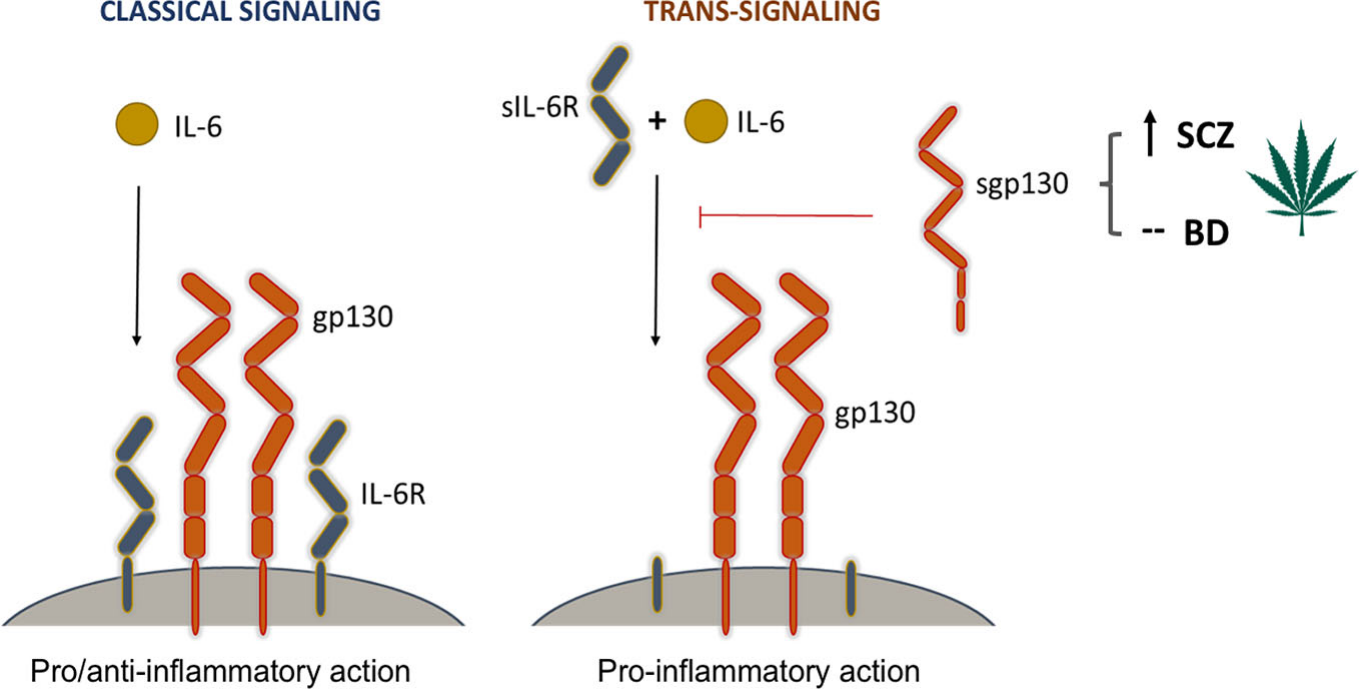
The modulatory effect of *Cannabis* on signaling pathways mediated by gp130. Interleukin-6 (IL-6) is a central player in both pro-inflammatory and anti-inflammatory immune responses. It can modulate cellular responses in two ways. In classical signaling, it binds to its cell membrane-bound receptor (IL-6R) and triggers a heterodimeric association with two membrane-bound gp130 molecules. This signaling complex initiates downstream pro- and anti-inflammatory responses through the activation of three signaling cascades, most prominently the JAK-STAT pathway. In trans-signaling, IL-6 binds to the soluble form of its receptor (sIL-6R) before it forms a complex with membrane-bound gp130. This complex then activates downstream pathways leading to pro-inflammatory responses. Soluble gp130 (sgp130) has an inhibitory effect on trans-signaling by blocking the association of the IL-6/sIL-6R complex with membrane-bound gp130 molecules. *Cannabis* use, which was found to increase the levels of sgp130 in schizophrenia patients, is thus believed to have a suppressing effect on inflammation by inhibiting the initiation of trans-signaling cascades.

The critical importance of the regulation of IL-6 trans-signaling through the blood-brain-barrier has been demonstrated in central nervous system inflammation, as well as in neurodegenerative and neuropsychiatric disorders, such as Alzheimer’s and Parkinson’s [reviewed in (22, 23)]. The regulatory effect of gp130 on inflammation is tightly linked to the pleiotropic nature of the IL-6 system in mammals. Gp130, which exists in both a membrane-bound form (gp130) and in a soluble form (sgp130), is a common signal-transducing subunit of several cytokine receptors involved in inflammation and immunity including but not limited to IL-6 (24). Although IL-6 can be produced by several immune and non-immune cells, its receptor (IL-6R) expression is restricted to only a subset of cells, such as certain lymphocytes (25), microglia (26), and hepatocytes (27). IL-6R also exists in a soluble form (sIL-6R) produced *via* alternative splicing or limited proteolysis (23). Signaling of the IL-6/IL-6R complex requires the cell membrane-bound form of the co-receptor gp130. Binding of the complex to gp130 initiates downstream signaling targeting JAK2/STAT3, PI3 kinase, ERK, and other intracellular effector pathways (28). IL-6 can modulate cellular functions in two different ways (**Figure 3**): In classical signaling, IL-6 binds to IL-6R and then the complex triggers the dimerization of membrane-gp130 leading to downstream effects that are pivotal in the regulation of anti-inflammatory signals (29). In IL-6 trans-signaling, IL-6 is bound by sIL-6R and the complex can activate distant cells expressing membrane-bound gp130 in a paracrine and endocrine manner which is dominantly pro-inflammatory (23). Gp130 signaling is also involved in neuronal and astrocyte differentiation and survival (30), and in the astrocytic control of CNS neurotoxic inflammation (31, 32).

Soluble gp130 is a decoy receptor that is produced under physiological conditions and is able to bind the IL-6/sIL-6R complex and thereby potently and selectively blocking inflammatory trans-signaling (**Figure 3**) (28, 33). Thus, it can alleviate the harmful *in vivo* effects of IL-6 in the CNS (34). Indeed, sgp130 has been shown to exert strong anti-inflammatory effects in both local and systemic contexts (22, 23, 34, 35), and to protect from the effects of lipopolysaccharide (LPS)-induced sickness behavior in a murine model accompanied by dramatic decrease in IL-6 mRNA and protein levels in the hippocampus (35).

In accordance with the immune hypothesis of SCZ, dysregulated and/or low-grade chronic brain inflammation may interfere with cognition, mood regulation, and higher-order functioning, and any means of modulating this “inflammatory immune-brain” cross-talk may pose a potential therapeutic target (11, 12). In a recent study, sgp130 levels were found to be lower in SCZ and BD patients than in healthy controls (10). We hypothesize that *Cannabis* consumption may contribute to elevated levels of sgp130 in SCZ patients favoring a systemic anti-inflammatory milieu. This effect may be more pronounced if it is also accompanied by mild increase in the levels of the anti-inflammatory regulators IL-1RA and sTNFR1. Supportive of this idea, a recent report revealed that *Cannabis* use disorder is associated with decreased risk of diseases of gut-brain interaction and inflammatory bowel disease in SCZ, but not in healthy controls (36). Since circulating inflammatory biomarker levels are inversely correlated with negative symptom severity in SCZ (11, 12), the documented anti-inflammatory effect of *Cannabis* may stand in the background of the observed associations between *Cannabis* consumption and improved mood regulation.

The observed sgp130-modulating effect of *Cannabis* in SCZ may be due to its immunoregulatory potential, especially its effect on IL-6 signaling and presumably on IL-6 trans-signaling feedback-loops as suggested by others (13). The beneficial effects of *Cannabis* consumption in people with SCZ (“*Cannabis* self-medication hypothesis”) could be attributed to CBD, its natural anti-inflammatory and antipsychotic component, which has recently been reported to be clinically effective in the adjunctive therapy of SCZ (37, 38). Due to its observational nature, our study does not establish a causal link between *Cannabis* use and symptom severity or cognitive performance in SCZ, but may reveal a disease-specific phenomenon that is not present in BD. Additionally, the lack of temporal resolution with regard to our *Cannabis* data (i.e., the lack of control of timing relative to last *Cannabis* use) poses a major limitation to this study. Blood concentrations of the identified immune markers are expected to be affected by both the frequency and amount of *Cannabis* use, as well as the time span that has elapsed since last exposure. The fact that temporal differences were not accounted for, could explain why we did not see a dose-dependent relationship between sgp130 levels and *Cannabis* use after stratification by severity of use (use without disabilities, abuse, and addicted). Therefore, although our findings suggest an anti-inflammatory effect of sgp130 that may contribute to the therapeutic value of *Cannabis* in SCZ, elucidation of the physiological details requires future investigations, preferably by comparing sgp130 levels directly with urine concentrations of cannabinoids and their metabolites in SCZ and BD patients.

The absence of the inflammation-modulatory phenomenon of *Cannabis* in BD may resonate with earlier reports on the neutral or potentially adverse effects of *Cannabis* use on BD symptoms and disease evolution (39, 40). Furthermore, we speculate that our findings regarding the effect of *Cannabis* self-administration on circulating immune marker levels likely reflect the relatively high CBD content of available *Cannabis* products in the streets of Norway (41). On the other hand, the impact of the psychotropic cannabinoid THC on human brain functions has been highly controversial. It is important to note that, despite its general anti-inflammatory effect, exposure to THC, particularly during adolescence, is hypothesized to perturb the neuroendocrine-immune homeostasis evoking latent vulnerability to psychosis or exacerbate symptoms in individuals with SCZ (42–44). In agreement with this, a recent GWAS study involving a large cohort of lifetime *Cannabis* users revealed causal positive influence between *Cannabis* use and SCZ risk (45). Thus, while medical *Cannabis* and cannabinoids are emerging as potential therapeutic adjuvants in neuropsychiatric disorders and other diseases, it is very important to make efforts estimating the possible risks and benefits of cannabinoids in order to provide reliable and competent clinical care.

Taken together, our results show for the first time that *Cannabis* self-administration is associated with elevated levels of sgp130 and several other immune and neuroendocrine markers in SCZ, but not in BD, and that this phenomenon is independent of the modulation of peripheral immune cells. These findings warrant further investigation into the potential neuroimmune, anti-inflammatory, and biobehavioral-cognitive effects of *Cannabis* use in SCZ.

## Data Availability Statement

The datasets generated for this study are available on request to the corresponding author.

## Ethics Statement

The studies involving human participants were reviewed and approved by Norwegian Scientific Ethical Committee and the Norwegian Data Protection Agency. The patients/participants provided their written informed consent to participate in this study.

## Author Contributions 

Conceived and designed the study: AS, IA, and SD. Performed the statistical analyses: AS, IA, TU, TL, and ID. Clinical assessment/sampling/analysis tools: TU, TL, ID, IM, OA, and SD. Database curation and data analysis: AS, IA, TU, TL, ID, and TB. All authors contributed to the article and approved the submitted version.

## Conflict of Interest

The authors declare that the research was conducted in the absence of any commercial or financial relationships that could be construed as a potential conflict of interest.
